# cTBS over ventral cortex enhances depth perception

**DOI:** 10.3389/fnins.2024.1499030

**Published:** 2024-12-03

**Authors:** Justin K. N. Or, Dorita H. F. Chang

**Affiliations:** ^1^Department of Psychology, The University of Hong Kong, Pokfulam, Hong Kong SAR, China; ^2^The State Key Laboratory of Brain and Cognitive Sciences, The University of Hong Kong, Pokfulam, Hong Kong SAR, China

**Keywords:** repetitive transcranial magnetic stimulation, continuous theta burst stimulation, stereoscopic plasticity, occipital cortex, lateral occipital complex

## Abstract

Stereoscopic capacities vary widely across the normal population. It has become increasingly apparent, however, that mechanisms underlying stereoscopic depth perception retain a considerable degree of plasticity through adulthood. Here, we contrast the capacity for neurostimulation in the form of continuous theta-burst stimulation (cTBS) over strategically-chosen sites in the visual cortex to bring about improvements in stereoscopic depth perception. cTBS was delivered to occipital cortex (V1/V2), lateral occipital complex (LOC), along with a control site (Cz). We measured performance on depth and luminance discrimination tasks before and after stimulation. We found a significant improvement in depth (but not luminance) discrimination performance following cTBS over LOC. By contrast, cTBS over occipital cortex and Cz did not affect performance on either task. These findings suggest that ventral (lateral-occipital) cortex is a key node for governing plasticity of stereoscopic vision in visually normal human observers. We speculate that cTBS exerts inhibitory influences that may suppress internal noise within the nervous system, leading to an improved read-out of depth features.

## 1 Introduction

Stereopsis refers to the ability for the brain to resolve slight differences in the projections of images onto the left and right retinas, in order to form a representation of depth. Stereoscopic vision is valuable for interacting with the environment, allowing individuals to distinguish objects, estimate distances, and manipulate them effectively. As such, deficits in this capacity, as can be seen in individuals with binocular dysfunction (e.g., those with amblyopia (Levi et al., 2015)), may lead to struggles with every-day visuomotor tasks.

In recent years, an ever-growing body of work has focused on developing methods that promote neuroplastic changes in the visual system. In this vein, one technique gaining traction is brain stimulation. Recent studies have demonstrated that offline repetitive transcranial magnetic stimulation (rTMS) over the primary visual cortex (V1) can facilitate a wide range of visual functions, including contrast sensitivity and orientation discrimination (Thompson et al., 2008; Waterston and Pack, 2010). Since V1 presents the first emergence of binocular neurons, stimulation over V1 may also facilitate the analysis of depth positions. Indeed, Tuna et al. (2020) have shown that continuous theta burst stimulation (cTBS; Huang et al., 2005) over V1, despite its posited inhibitory effect on cortical excitability, improves a wide range of visual capacities in amblyopic adults, including visual acuity, suppressive imbalance, and most notably, stereoacuity.

Whether the beneficial effects of cTBS on stereovision are restricted to stimulation over V1 is unknown. There is no particular reason to assume that V1 stimulation should produce the most optimal benefits to stereopsis. Neurophysiological studies have shown that the computation of binocular disparity begins from V1 and extends to dorsal and ventral visual regions that are engaged in a task-dependent manner (Preston et al., 2008; Tsao et al., 2003; Uka and DeAngelis, 2006). Specifically, ventral areas including V4, lateral occipital complex (LOC), and inferior temporal cortex (IT), are engaged during fine discriminations of stereo-features (Preston et al., 2008; Shiozaki et al., 2012; Uka et al., 2005). In particular, previous work has shown that LOC activation reflects the categorical distinction between “near” and “far” depth positions in a fine depth discrimination task (Preston et al., 2008). Human lesion studies also revealed that cortical damage in the LOC interrupts fine disparity discrimination (Read et al., 2010). Using a disruptive online rTMS protocol, Chang et al. (2014) similarly revealed that LOC stimulation selectively hampers fine stereoscopic performance in healthy vision, and that its relevance changes following perceptual training. Given the prominent involvement of LOC in fine depth judgments, it might yet be a more sensible locus to modulate in an attempt to enhance stereovision.

We investigated here whether cTBS over LOC can bring about improvements in stereoscopic depth perception. Specifically, we tested the specificity of cTBS-induced effects (if any) to stimulation site and to visual feature (are improvements depth-specific?). Establishing the feature/task-specificity of cTBS-induced effects at the various stimulation sites will be particularly revealing given previous work showing the breadth of improvements attainable following V1 stimulation (Tuna et al., 2020), at least in Amblyopes. We expect, on the contrary, that ventral stimulation may not produce the same generalized improvements across features given its putative specialization for form and shape, but also to stereo (Chang et al., 2014; Grill-Spector et al., 2001; Kourtzi et al., 2003).

We tested three stimulation sites (early occipital cortex, V1/V2; LOC; and control site vertex, Cz). We also tested two tasks: a fine stereo-depth discrimination task and a secondary luminance discrimination task. While in principle, we could have selected any secondary feature in order to probe the specificity of cTBS-induced effects at our stimulation sites, we opted for a luminance discrimination task as it carries a feature generally processed very early in the visual cascade (Goodyear and Menon, 1998; Albrecht and Hamilton, 1982), and can be judged devoid of fine form or features that would otherwise engage well-established lateral-occipital areas (Avidan et al., 2002; Grill-Spector et al., 1999; Malach et al., 1995). We compared performances on depth and luminance discrimination tasks before and after stimulation over early occipital cortex (V1/V2), LOC, and control site vertex (Cz). In the depth task, participants were asked to discriminate between small depth positions (Chang et al., 2014; Chang et al., 2013). In the luminance task, stimuli were designed analogously to those in the depth task, but participants were instructed to ignore the depth positions and to instead judge the luminance differences in the stimulus.

## 2 Materials and methods

### 2.1 Participants

A total of 52 normal-sighted adults (age 18–37 years, mean 21.8 years; 20 males) participated in the current study. An additional 8 participants were recruited but failed to complete the tasks (i.e., initial discrimination thresholds at ceiling, see Stimuli and Tasks below). All participants were tested to have normal or corrected-to-normal visual acuity (LogMAR chart), stereoacuity (Butterfly test), and binocular fusion (Worth-4-Dot test). Participants were additionally screened for contraindications to TMS. Exclusion criteria included abnormal vision, a history of neurological and psychiatric disorders, a family history of seizures, or the presence of metal implants (Wassermann, 1998; Rossi et al., 2021). Participants were asked not to ingest caffeine (an hour) and alcohol (a day) before the test and to have enough sleep (more than 6 h) before attending the session, due to their known effects on cortical excitability and TMS measures (Turco et al., 2020; Civardi et al., 2001). Written informed consent was obtained from all participants. The experimental procedures were approved by the Human Research Ethics Committee of The University of Hong Kong. Participants were randomly assigned to three groups: 17 received occipital stimulation (9 left V1/V2; 8 right V1/V2), 18 received LOC stimulation (9 left LOC; 9 right LOC), and 17 received Cz stimulation. One participant was reassigned from the occipital stimulation group to receive Cz stimulation due to the inability to understand instructions for perceiving and/or inability to perceive phosphenes. Remuneration was offered in the form of monetary compensation or course credits.

### 2.2 Apparatus

Participants were tested in a dimly lit room. The viewing distance was maintained at a distance of 50 cm by means of a chinrest. Stimuli were generated using custom software written in MATLAB (version R2019a; MathWorks, Natick, MA, USA) with extensions from Psychophysics Toolbox (Brainard, 1997; Pelli, 1997), running on a PC equipped with a pair of shutter goggles (NVIDIA 3D Vision 2 Wireless; NVIDIA Corporation, Santa Clara, CA, USA). Stimuli were displayed on an ASUS VG278QF 27-inch monitor (ASUSTek Computer Inc. Taipei, Taiwan; spatial resolution: 1920 × 1,080 pixels; refresh rate: 120 Hz) equipped with an infrared emitter.

### 2.3 Procedure

The entire protocol comprised four phases: practice, pre-stimulation tests, cTBS stimulation delivery, and post-stimulation tests. These phases were conducted consecutively on the same day. In the practice phase, participants completed 30 trials for each task with auditory feedback to get familiarized with the task. Participants then completed pre-stimulation tests with no auditory feedback, each consisting of two blocks of trials (for the fine depth task) and one block of trials (for the fine luminance task). The order of the tasks was counterbalanced among participants. Participants were allotted a 1-min break between the completion of each task. In the stimulation phase, a train of cTBS was applied to the targeted site. The total duration of the stimulation phase (including site localization and stimulation delivery) was around 15 min. Upon completion of cTBS, participants were allotted a 2-min break, after which they completed the post-stimulation test phase (in the same task order as that assigned in the pre-stimulation test phase).

### 2.4 Transcranial magnetic stimulation

Stimulation was administered with a Magstim Rapid^2^ Plus Stimulator (Magstim, Withland, Wales, UK), paired with a 70 mm figure-of-eight coil. All participants underwent an offline delivery of cTBS over 40 s (Huang et al., 2005). For this procedure, a total of 600 pulses was applied continuously in 5-Hz bursts which consisted of three pulses at 50 Hz. A fixed stimulation intensity was employed for all participants and stimulation sites (Waterston and Pack, 2010; Clavagnier et al., 2013; Kaderali et al., 2015). We chose not to use phosphene thresholding to obtain individualized stimulation *intensities* because TMS over the LOC rarely induces reliable phosphenes (Schaeffner and Welchman, 2017), and there is no evidence that the phosphene thresholds obtained from occipital stimulation would be appropriate for LOC stimulation, given variations in cortical density and skull thickness. Hence, we fixed the stimulation intensity at 50 percent of maximum device output—the average intensity at which previous findings have shown to be effective, when delivered as cTBS over V1, for inducing stereoscopic improvements in amblyopes (Tuna et al., 2020). During stimulation, participants were instructed to keep their eyes closed and to minimize their head movement. The coil was held over the scalp tangentially with the handle pointing upward for occipital and LOC stimulation or pointing posteriorly for Cz stimulation.

### 2.5 Stimulation sites

**Figure 1:**
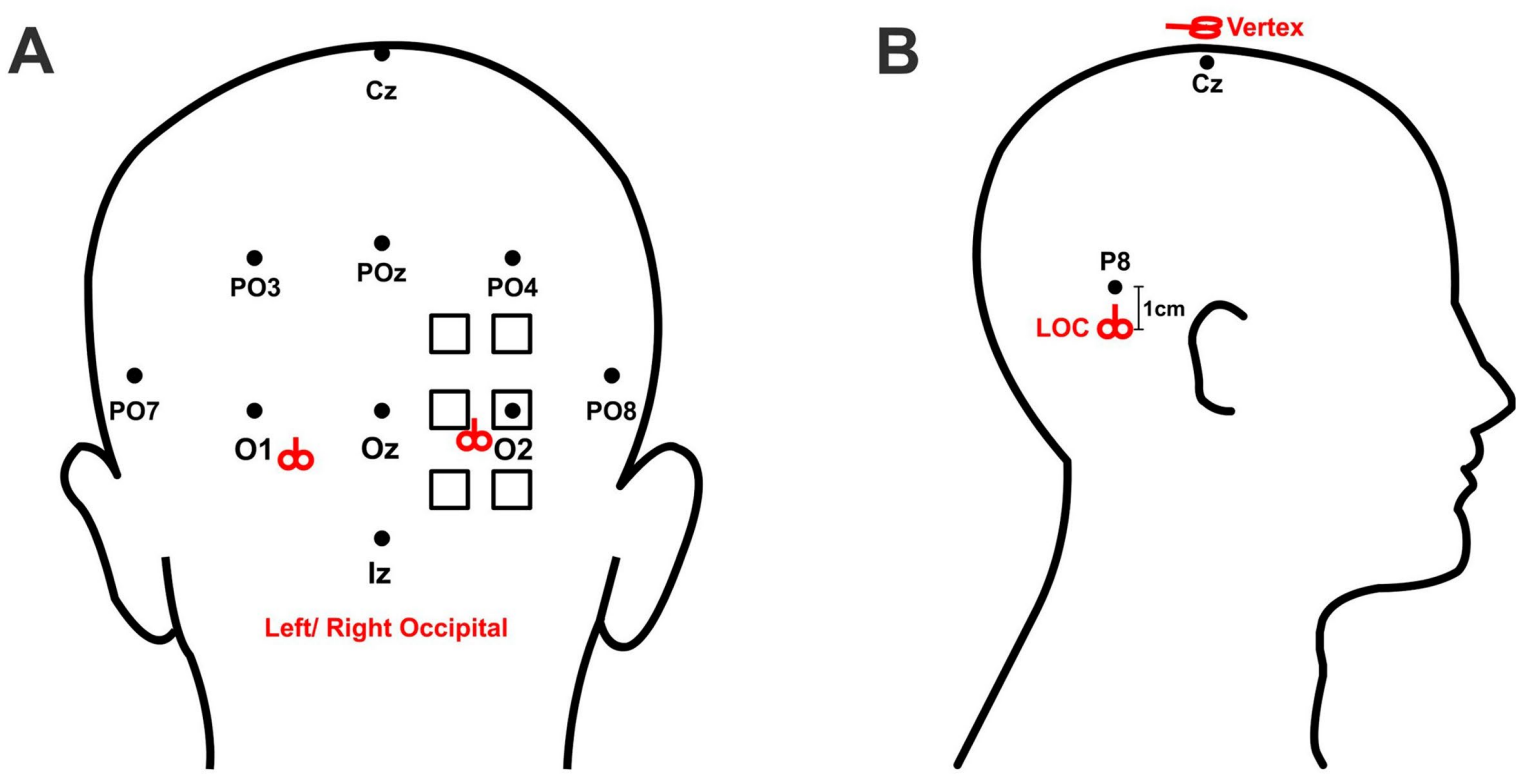
Localization of stimulation sites. The standardized 10–10 EEG coordinate system was used for localizing stimulation targets. **(A)** The six possible stimulation sites for right occipital stimulation are marked by the squares. The final stimulation site was defined by the location which elicited the strongest phosphene. For left occipital stimulation, corresponding regions on the left hemisphere were stimulated. The coil handle pointed upward for occipital stimulation. The right and left mean locations of occipital stimulation are marked by the red symbols of coil. **(B)** LOC stimulation was applied 1 cm below position P8 (for left LOC) or P7 (for right LOC). Control stimulation was applied over the vertex, defined as position Cz. The coil handle pointed upward for LOC stimulation and posteriorly for Cz stimulation.

To assist with identifying stimulation sites, participants wore a TMS-compatible 10–10 EEG stretch cap (Neuroelectrics Barcelona, SLU). The cap size was selected according to participants’ head circumference to ensure that the cap (and therefore the 10–10 coordinates) suited their head well. The cap position was adjusted so that position Cz was located at the midpoint between the glabella (the area between the eyebrows) and the inion (the most prominent point of the external occipital protuberance). For the occipital stimulation group, participants were randomly assigned to receive cTBS over the left or right V1/V2. The exact stimulation site for V1/V2 on each subject was localized through phosphene induction, a commonly used protocol for determining the optimal stimulation position over visual cortex (Tuna et al., 2020; Clavagnier et al., 2013). Specifically, we applied a pair of pulses at 20 Hz at 80% of maximum device output (Clavagnier et al., 2013; Boroojerdi et al., 2000) to six predetermined locations, arranged in a 2 × 3 grid, with each site spaced 1 cm apart from its adjacent neighbors and the central site in the lateral column aligned with position O1/O2 (Figure 1A). Since the phosphenes induced by stimulation are subjectively faint and transient, an eye patch was employed to occlude participants’ eyes to avoid extraneous visual interference. V1/V2 was defined as the location that elicited the brightest phosphene according to participants’ descriptions. For the LOC stimulation group, participants were randomly assigned to receive cTBS over the left or right LOC, located 1 cm below position P7 or P8, respectively. For these ventral positions, while P7 and P8 of the 10–10 coordinate system have been thought to broadly overlay the inferior temporal gyri (Koessler et al., 2009), its projected anatomical centroids nevertheless fall superior to the large swath of cortex typically reported to constitute the functionally-localized LOC, including regions of LO proper (along the posterior inferior temporal sulcus) and pFS in the posterior fusiform (Kourtzi et al., 2003). Hence, we elected to position our stimulation slightly below these landmarks. For the control group, cTBS was applied over the vertex, position Cz. The stimulation positions are illustrated in Figure 1B.

### 2.6 Stimuli and tasks

Stimuli in both tasks were designed to have similar configurations but were different in terms of their task requirements. Stimuli consisted of random-dot stereograms (RDS) depicting a central circular target plane (4.5° in diameter) surrounded by an annulus (9° in diameter). The stimuli were presented on a mid-gray background (luminance = 1.96 cd/m^2^). Dots of the RDS were 0.2° in size and had a density of 12 dots/degree^2^. The RDS’ were additionally surrounded by a binocularly presented grid of black and white squares 1.5° × 1.5° in size, designed to aid stable vergence as an unambiguous background reference. On each trial, participants maintained their gaze on a central nonius fixation. Task stimuli in the current experiment were presented centrally as eye tracking data in our pilot experiment indicated that lateralized stimulus presentation frequently led to the failure of fixation maintenance (side glancing towards the stimulus). Nonetheless, previous work has shown robust TMS effects using lateralized stimulation with centrally presented stimuli (Clavagnier et al., 2013; Allen et al., 2014).

For both tasks, a block of trials consisted of two interleaved staircases of 52 trials (104 trials in total). Task difficulty (disparity and luminance difference) was adjusted by the QUEST adaptive staircase procedure yielding thresholds at an 82% correct rate (Watson and Pelli, 1983). The staircases allowed reliable estimation of psychophysical thresholds in a timely manner as necessitated by the transient nature of TMS effects. Stimuli were presented for 300 ms after which a response was allowed during a fixed inter-stimulus interval of 1,700 ms. Participants responded by pressing the arrow keys on a computer keyboard.

#### 2.6.1 Depth task

**Figure 2:**
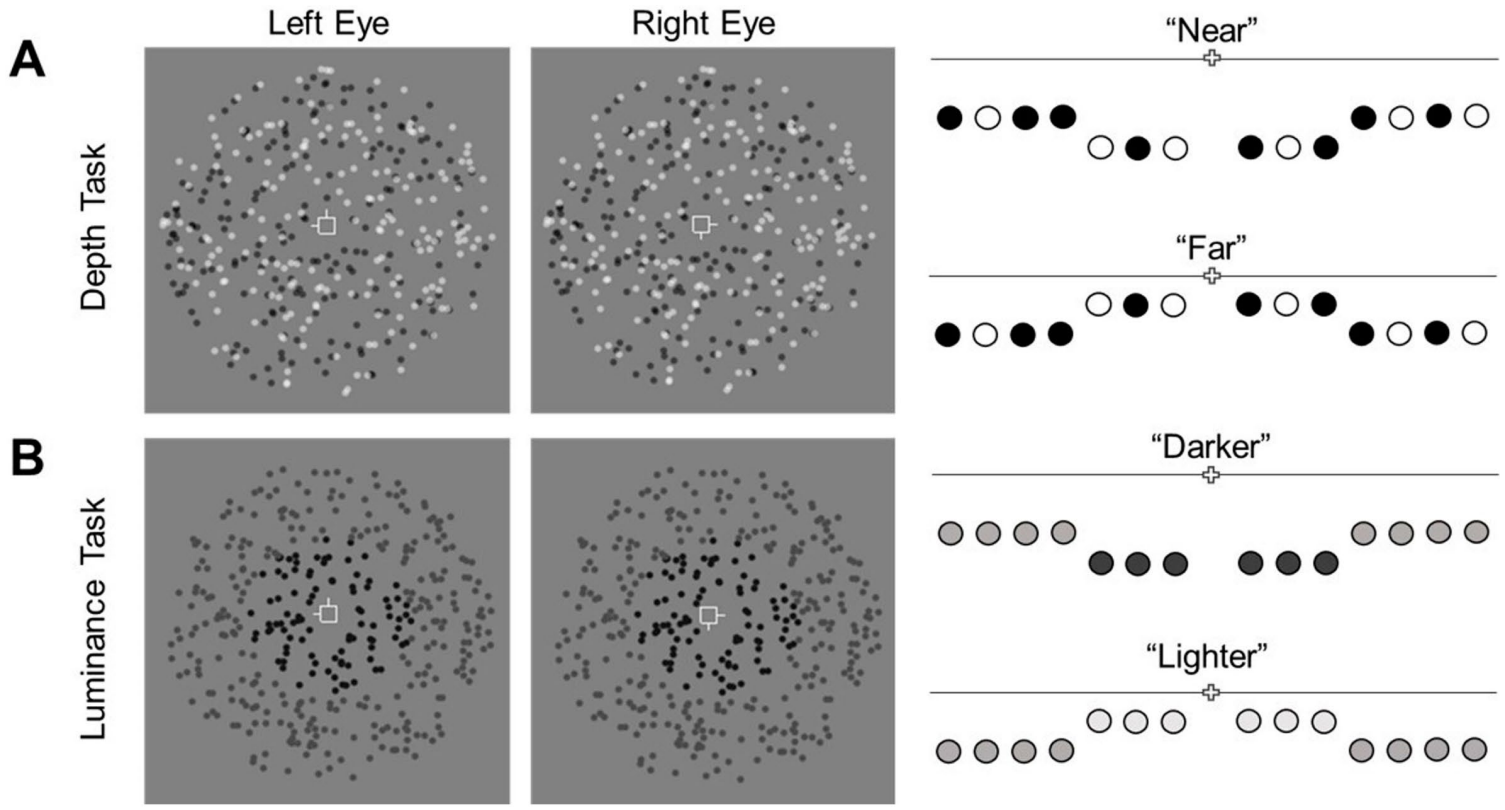
Illustration of the discrimination tasks. Participants fixated on a central nonius fixation. **(A)** In the depth task, the disparity of dots in the central plane varied finely across trials and was either “near” or “far” relative to the surrounding annulus. Participants judged the disparity difference between regions. **(B)** In the luminance task, the luminance of the central dots varied relative to the surrounding dots. Participants judged whether the central dots were darker or lighter than the surround dots.

In the depth task, all dots of the RDS’ were randomly black or white in equal proportion. On each trial, participants judged whether the central target plane was in front of (“near”) or behind (“far”) the surround (Figure 2A). The disparity of the surrounding plane was fixed at 720 arcsec. The disparity difference between the two planes varied across trials (1–360 arcsec). Each staircase comprised an equal number of “near” and “far” stimuli presented in random order. Depth performance was computed as the average of thresholds obtained from the two staircases.

#### 2.6.2 Luminance task

In the luminance task, all dots of the RDS’ were presented in uniform gray-scale luminance. The luminance of dots in the surround was fixed at 0.68 cd/m^2^. The luminance difference between central and surrounding regions varied across trials with a maximum of ±0.86 cd/m^2^. This range was designed to maintain the discriminability between the dots of the RDS’ and the mid-gray background (luminance = 1.96 cd/m^2^). Similar to the depth task, the two regions also differed in disparity (although it was task irrelevant). The disparity of the surrounding plane was fixed at 720 arcsec while the disparity of the central target plane was fixed at ±47.91 arcsec from the surround. This disparity difference was determined from pilot data (thresholds) obtained for the depth task. In each trial, participants indicated whether the central target plane was darker or lighter than the surround (Figure 2B). Equal proportions of “near” and “far,” and, “darker” and “lighter” trials were randomly presented in each staircase. Luminance discrimination performance was computed as the mean threshold estimates of the two staircases.

## 3 Results

**Figure 3:**
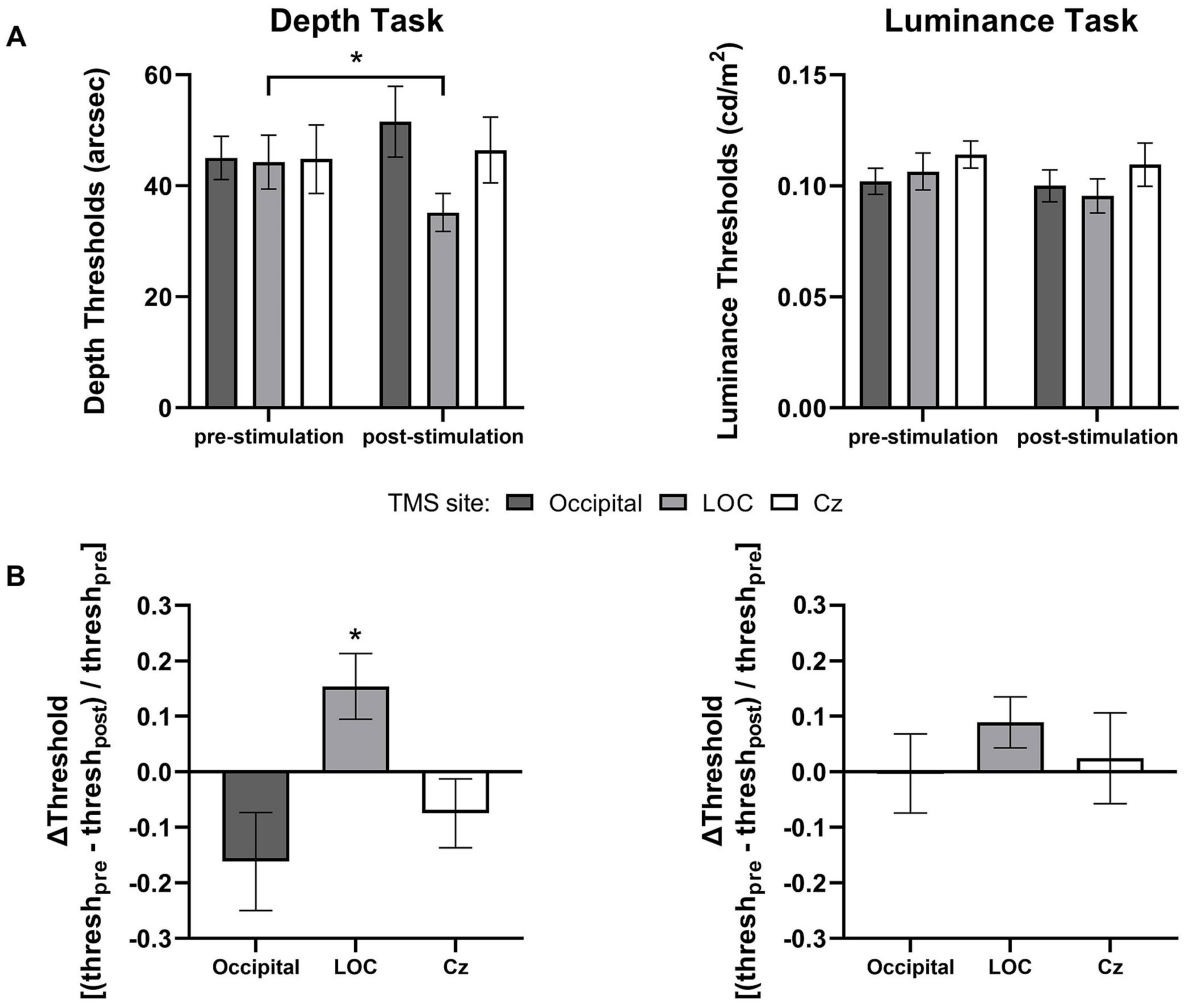
Stimulation-induced changes in task performance. **(A)** Mean thresholds for the depth and luminance discrimination tasks before and after stimulation over occipital cortex (*n* = 17), LOC (*n* = 18), and Cz (*n* = 17). **(B)** The data are re-presented as normalized changes in pre-stimulation versus post-stimulation thresholds (i.e., pre-post/pre) in the depth and luminance tasks. Error bars represent ±1 SEM. *p < 0.0167.

Thresholds for the depth and luminance tasks are presented for the different stimulation groups and across the pre and post-tests in Figure 3A. To facilitate visual comparisons, the data were replotted in terms of normalized thresholds (pre-post/pre) in Figure 3B. See also Supplementary Figure 1 for an overlay of individual subject performance, before and after stimulation, for both the depth and luminance tasks.

To rule out the possibility that changes in performance, if any, were caused by differences in baseline performance among groups, we first compared pre-stimulation thresholds across the stimulation groups. Two one-way ANOVAs (one for each task) showed that there was no significant difference in baseline disparity [*F*_(2,49)_ = 0.006, *p* = 0.994, *η*^2^_p_ < 0.001] or luminance discrimination thresholds [*F*_(2,49)_ = 0.76, *p* = 0.473, *η*^2^_p_ = 0.03] across the stimulation groups.

We then assessed the difference in disparity discrimination thresholds before and after stimulation across groups by means of a 3 (group) × 2 (test: pre versus post) mixed ANOVA. The analysis indicated no significant main effects of group [*F*_(2,49)_ = 0.813, *p* = 0.449, *η*^2^_p_ = 0.032] and test [*F*_(1,49)_ = 0.021, *p* = 0.884, *η*^2^_p_ < 0.001], but a significant group × test interaction [*F*_(2,49)_ = 5.131, *p* = 0.009, *η*^2^_p_ = 0.173]. Bonferroni corrected paired *t*-tests revealed that depth thresholds significantly improved (i.e., dropped) after LOC stimulation [*t*_(17)_ = 2.66, *p* = 0.0166], but not after occipital [*t*_(16)_ = 1.48, *p* = 0.158] or Cz stimulation [*t*_(16)_ = 0.63, *p* = 0.538]. In particular, LOC stimulation improved depth discrimination thresholds by 20.5% (from 44.28 to 35.19 arcsec) at the group level (Figure 3).

We further compared performance changes induced by left vs. right LOC stimulation. Although the average improvement in disparity discrimination thresholds following right LOC stimulation (21.7%) was slightly larger than that following left LOC stimulation (19.3%), a 2 (hemisphere) × 2 (test) mixed ANOVA only indicated a significant main effect of test [*F*_(1,16)_ = 6.682, *p* = 0.020, *η*^2^_p_ = 0.295]. There was no significant effect of hemisphere [*F*_(1,16)_ = 0.303, *p* = 0.590, *η*^2^_p_ = 0.019] nor hemisphere × test interaction [*F*_(1,16)_ = 0.094, *p* = 0.763, *η*^2^_p_ = 006]. That is, improvements following cTBS over left and right LOC did not differ significantly.

Luminance discrimination thresholds were entered into a separate 3 (group) × 2 (test) mixed ANOVA. The results revealed no significant difference in luminance thresholds across groups [*F*_(2,49)_ = 0.868, *p* = 0.426, *η*^2^_p_ = 0.034] and tests [*F*_(1,49)_ = 1.905, *p* = 0.174, *η*^2^_p_ = 0.037]. There was also no significant group × test interaction, *F*_(2,49)_ = 0.400, *p* = 0.672, *η*^2^_p_ = 0.016 (Figure 3).

## 4 Discussion

We tested participants’ performance in two tasks carrying analogous stimuli, but differing task demands, before and after cTBS over V1/V2, LOC, or Cz. We found that stimulation over LOC, but not over occipital cortex nor control site Cz, improved participants’ capacity to discriminate between depth positions (“near” or “far”). Stimulation-induced improvements in depth discrimination did not differ depending on the laterality of brain stimulation, although right LOC stimulation tended to induce greater improvements. These findings are in line with previous studies that have identified critical computations in LOC during depth judgments—namely, reading-out and grouping low-level visual features, representing global object structure (Grill-Spector et al., 2001; Murray et al., 2002), and discriminating near-far depth positions (Preston et al., 2008; Read et al., 2010; Chang et al., 2014).

In contrast to the changes observed for the depth task, we found that stimulation over all three sites did not elicit reliable changes in performances on the luminance discrimination task. Hence, the perceptual improvements observed following cTBS over LOC are specific to stereoscopic depth perception and do not generalize to the perception of other visual features (or at least, to the luminance feature tested here). These particular findings are in alignment with previous work that have demonstrated that LOC activations are invariant to low-level visual features and have a low contrast dependence (Avidan et al., 2002; Grill-Spector et al., 1999). That is, changes in luminance contrast do not affect the representation of object shape and identity (i.e., luminance and contrast-invariant).

An obvious curious question that follows is: how is cTBS driving enhancements in depth perception? Psychophysical performance hinges on the interplay between the sensory signals and background neuronal noise. While cTBS-induced cortical inhibition may decrease the overall activity of cortical neurons, the suppression of neuronal noise could offset the reduced signal-related activity (Allen et al., 2014). As noise arises from random fluctuations in neuronal activity (Kohn et al., 2016; Tolhurst et al., 1983), a moderate increment in inhibition (delivered via cTBS) could significantly reduce the likelihood of spontaneous noisy discharge of neurons that do not represent any meaningful information (Allen et al., 2014). Alternatively, signal-related activity primarily stems from post-synaptic potentials, rendering it relatively less susceptible to cTBS-induced cortical inhibition. When neuronal noise exhibits correlated variability in firing rates from pairs of neurons, perceptual processing faces increased difficulty in distinguishing signal from noise (Zohary et al., 1994). Improvements following cTBS observed in previous studies have been attributed to noise decorrelation (Waterston and Pack, 2010; Clavagnier et al., 2013; Hamidi et al., 2009). In particular, a model put forth by Waterston and Pack (2010) provides a theoretical framework that demonstrates how noise decorrelation could outweigh the reduced firing rate due to inhibitory cTBS, ultimately leading to a better signal-to-noise ratio and more precise processing of sensory signals. In line with this, we conjecture that cTBS over LOC may enhance depth performance here by improving representations of the disparity-defined object shape (i.e., promoting readout) through noise suppression.

The specificity of cTBS-induced improvements to stimulation site observed here suggests further that cTBS-induced noise-reduction is region-specific. Previous work probing the effects of perceptual learning and attention have reported that their behavioral consequences on noise decorrelations vary across regions. Although both learning and attention have often been found to reduce noise correlations, noise decorrelations in different areas do not always benefit population coding efficiency (Sanayei et al., 2018). For example, training-induced and attention-spotlit noise decorrelations do not benefit fine orientation discriminations in V1 nor in the dorsal medial superior temporal area (MSTd; Gu et al., 2011; Ni et al., 2018). However, they correlate well with improvements in orientation and contrast discriminations in V4 (Sanayei et al., 2018; Ni et al., 2018). Despite the obvious differences in task and paradigms between those studies and ours, we observed similarly that cTBS over ventral area LOC, but not occipital cortex (V1/V2), enhanced depth discrimination. As such, it is possible that the noise decorrelations in LOC exclusively augment the population coding efficiency pertaining to depth discrimination.

The lack of consequences observed with occipital stimulation may seem surprising in light of several previous studies showing that occipital stimulation enhances various visual capacities in visually normal adults (Waterston and Pack, 2010; Allen et al., 2014; Maniglia et al., 2019; Schaeffner and Welchman, 2019). Remarkably, our results stand in contrast to previous work that reported fine stereoscopic improvements in amblyopic adults following cTBS over phosphene-defined V1 (Tuna et al., 2020). The discrepancy between these findings and those of ours suggest perhaps that the effects of occipital cTBS on stereoscopic depth perception vary with the population tested. While occipital cTBS improves stereoacuity in amblyopic vision through yet unknown mechanisms, it is apparently not effective in healthy vision. Why might this be?

One clear difference between the amblyopic and healthy visual systems concerns their baseline stereoacuity. The baseline stereoacuity levels reported by Tuna et al. (2020) were extraordinarily high (with a median value of 100 arcsec and a mean value of 258.3 arcsec) compared to the average baseline recorded in our behavioral experiment (44.7 arcsec). Thus, it seems possible that occipital stimulation, while effective, manifests more evidently in those with poorer baseline stereovision. This baseline-dependent characteristic of effects elicited by occipital stimulation has also been reported in previous studies across various visual capacities (Thompson et al., 2008; Silvanto et al., 2018).

Amblyopic vision is thought to be hindered by anomalous binocular interaction in the nervous system (among other monocular deficiencies; Holmes and Clarke, 2006). Due to anomalous visual experience in early development, the amblyopic brain chronically suppresses the visual input from the amblyopic eye (Harrad et al., 1996; Jampolsky, 1955). Along the visual hierarchy, interocular suppression first appears at or just before the emergence of binocular neurons in V1 (Baker et al., 1974; Kiorpes and McKeet, 1999). Therefore, it is entirely possible that there is a difference between amblyopes and in visually normally individuals in terms of the stage at which performance is limited: in amblyopic vision, this may well be V1, before the disparity information enters higher-order visual areas for subsequent stereoscopic analysis. In normal vision, our data appear to indicate that performance is limited well past occipital cortex, towards LOC. The relevance of ventral cortex to stereo performance in the normal visual system is supported by previous lesion and TMS evidence showing that disruption (or lesion) of LOC substantially worsened the fine disparity discrimination (Read et al., 2010; Chang et al., 2014).

Moving forward, one potential way to test the exact mechanistic changes elicited by cTBS, in particular as it pertains to noise reduction, is by examining the possibility of reversing the effects of LOC stimulation through elevating stimulation intensity. The logic is that a greater increment of inhibition may lead to more pervasive suppression which in turn disrupts the signal-readouts and thereby the perceptual judgment (Allen et al., 2014). It would be intriguing if LOC stimulation ends up producing a disruptive effect on stereopsis when stimulation intensity elevates beyond a certain point.

Moreover, although our data indicated that cTBS over LOC enhanced performance on the depth, but not luminance discrimination task, it may be beneficial to further discern whether this effect represents a general enhancement of all LOC-related capacities (Murray et al., 2002; Beauchamp, 2005) or represents a true, specific enhancement of depth perception. As noise permeates every level of information processing in the nervous system, it is possible that the reduction of noise in LOC also benefits other functions that rely highly on the LOC activations.

In summary, we showed that cTBS can improve disparity discrimination when LOC is targeted, suggesting that LOC is a critical locus for disparity processing, and is perhaps due for further attention for its neuroplastic capacities under (likely non-amblyopic-relevant) rehabilitative circumstances. Under the present conditions, individuals who instead received occipital stimulation did not show reliable changes in stereoscopic performance. This stands in contrast to previous work that has reported enhanced stereoacuity in amblyopic vision immediately following occipital stimulation. We therefore conjecture that stereoscopic depth perception is limited by mechanisms at different stages of the visual hierarchy in the amblyopic and healthy visual systems. Considering the posited inhibitory effects of cTBS, we speculate that cTBS enhances signal-to-noise ratio by suppressing internal noise in the nervous system thereby contributing to better read-outs of the disparity signal.

## Data Availability

The raw data supporting the conclusions of this article will be made available by the authors, without undue reservation.
