# Supplementary material for: cTBS over ventral cortex enhances depth perception
Source: Front Neurosci. 2024 Dec 3;18:1499030. doi: 10.3389/fnins.2024.1499030 (PMC11653416; doi:10.3389/fnins.2024.1499030)
Supplement: Supplementary file 1 [file Data_Sheet_1.docx]

Supplementary Material for “cTBS over ventral cortex enhances depth perception”

**
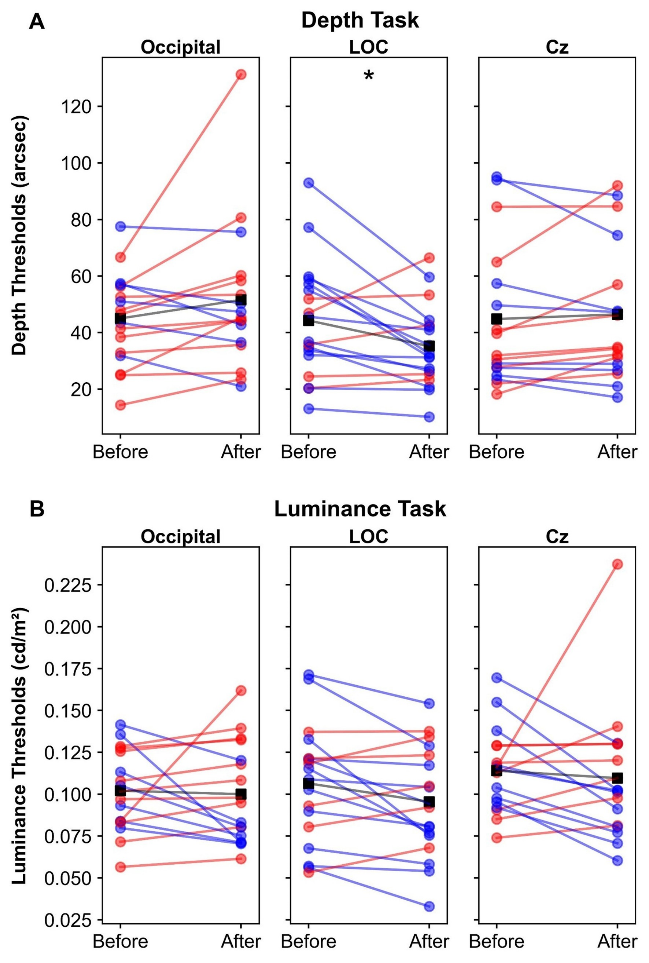
**

**Supplementary Figure 1. Pairwise Comparison of Discrimination Thresholds before and after cTBS.** Individual subject data corresponding to **(A)** depth and **(B)** luminance thresholds before and after cTBS over three regions of interest: occipital cortex (V1/V2), lateral occipital complex (LOC), and vertex (Cz). Lines connecting the markers show the changes for individual participants, where blue lines denote improvements and red lines denote poorer performances after stimulation. The black line represents the change in mean thresholds for each condition. ROI(s) with statistically significant changes are marked with an asterisk (*).
